# The Impact of Thermal Treatment and In Vitro Digestion on Antioxidant Activity and Anti‐Glycation Properties of Antioxidant Crude Extract From Hot and Cold Brew Spent Coffee Ground

**DOI:** 10.1002/fsn3.70131

**Published:** 2025-04-30

**Authors:** Onamon Chongsrimsirisakhol, Kamolwan Jangchud, Peter James Wilde, Tantawan Pirak

**Affiliations:** ^1^ Department of Product Development, Faculty of Agro‐Industry Kasetsart University Bangkok Thailand; ^2^ Quadram Institute Bioscience Norwich UK

**Keywords:** advanced glycation end product, antioxidant activity, antioxidant crude extract, cold brew spent coffee ground, hot brew spent coffee ground, in vitro digestion

## Abstract

The ethanoic extract of hot‐brew spent‐coffee ground (HSCG) and cold‐brew spent‐coffee ground (CSCG) were prepared with ultrasound‐assisted extraction and subjected to thermal processes and in vitro digestion prior to analyze the inhibition ability of advanced glycation end products (AGEs) formation, a potential risk factor for Alzheimer's disease. The obtained HSCG and CSCG extracts contained mainly chlorogenic acid derivatives, according to liquid chromatography‐mass spectrometer chromatogram. The glycation process was performed by using bovine serum albumin (BSA)/ glucose system with 3 weeks incubation. In the presence of HSCG and CSCG extracts at 250 μg/mL, after thermal treatment (pasteurization and sterilization) and in vitro digestion, the glycation process through the fructosamine, AGEs, and amyloid cross β structure formation was monitored, and these extracts exhibited an anti‐glycation property at early and advanced stages after pasteurization and in vitro digestion compared to no thermal treatment. However, at high temperature of sterilization, the suppress of anti‐glycation property had resulted and were related with the amount of antioxidant and the ability of antioxidant scavenging as presented in a dose manner. The calculated % caffeine bio‐accessibility of HSGC extracts was 65.8%, 64.8%, and 52.4% in non‐thermal, pasteurized, and sterilized samples while the higher bio‐accessibility was found in CSCG sample as of 67.4, 66.6, and 63.1, respectively. A high correlation of TPC values, thermal treatments and in vitro digestions with the AGEs was detected. Polyphenols and caffeine content in these extracts were found to be responsible for the AGEs and amyloid cross β structure inhibition which might potentially reduce the risk of Alzheimer's disease.

## Graphical abstract

CSCG extracts from the cold brew coffee process had a higher TPC, antioxidant activity, and caffeine content than HSCG extract from the hot brew coffee process. Thermal processing, especially sterilization, significantly reduced the TPC, antioxidant activity, and caffeine contents. In vitro digestion was found to be the mechanism that further reduced TPC, antioxidant activity, and caffeine content. A high correlation was detected with TPC values mirroring the effects of thermal treatments and in vitro digestions on the inhibition ability of the CSGC extract on glycation products and amyloid structure formation. Crude ethanoic extracts from HSCG and CSCG were by‐product value‐added compounds for inhibiting the formation of AGEs and amyloid cross β structure, which had the potential for risk reduction of Alzheimer's disease.

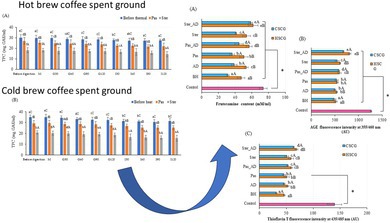

## 1 Introduction

Spent coffee grounds (SCGs) are the by‐product produced from every single coffee brewing technique (Yang et al. 2016). There are many brewing techniques all around the world that are mainly designed to match personal preference and convenience (Alves et al. 2017). To produce 1 ton of soluble coffee, the SCG was generated approximately 2–4.5 tons. The quantity of SCGs has been generated around 6 million tons (wet basis) worldwide annually (Zhao et al. 2024). Moreover, SCGs are reported as a high value biomass resource rich in bioactive compounds, for example, polyphenols, polysaccharides, protein, lipid, cellulose, and lignin (Ahmed et al. 2024; Zhao et al. 2024). The valorization of this biomass is an effective strategy; instead of landfill or discard as waste, the extraction of bioactive compounds for use as functional ingredients in foods, beverages, pharmaceutical products, and cosmetics was proposed to increase value and promote waste reduction in an environmentally friendly manner. Among various brewing techniques, one of the recently developed coffee brewing techniques is the cold brewing process. Cold (4°C) or room temperature (25°C–30°C) water is used as the solvent medium to extract the soluble coffee compounds from the roasted coffee grounds (Fuller and Rao 2017). During the roasting process, the natural phenolic compounds found in green bean coffees were partially destroyed, but other antioxidant compounds, such as melanoidins were formed. These could be possible to maintain or increase antioxidant activity of roasted coffee beans. Hence, light‐roasted coffee beans had higher antioxidants than other roasted degrees due to the greater polyphenol content (Vignoli et al. 2016). In fact, the process of cold brew takes several hours to extract the coffee; however, a smooth taste, large numbers of volatile compounds, and low acidity result, and it is reported to be less likely to irritate the stomach when compared to hot brewed coffee (Mestdagh et al. 2017). The total phenolic content (TPC) and antioxidant activity of coffee obtained from the hot brewing process and cold brewing process are reported (Rao and Fuller 2018). Moreover, a previous report revealed that the SCGs obtained from cold brewing process (CSCG) have higher TPC and antioxidant capacity when compared to hot brew spent coffee grounds (HSCG) from drip, Moka, and espresso brewing techniques (Chongsrimsirisakhol and Pirak 2022). Hence, CSCG is a prominent raw material that still contains valuable phytochemical compounds such as polyphenols and flavonoids.

During food processing, thermal treatment is important for preserving and maintaining the safety and quality of food products. The process could reduce foodborne pathogen contamination, inactivate endogenous oxidative enzymes (PPD and POD), and denature spoilage enzymes to prolong the shelf life of the product. However, a negative aspect has also been reported through loss, degradation, or dysfunction of certain desirable nutrients (thermally sensitive compounds) mostly caused by the oxidation process (Choi et al. 2006; Kim et al. 2021; Peanparkdee et al. 2020; Rodríguez‐Roque et al. 2015; Roy et al. 2007; Tomas et al. 2017). Moreover, this process could also initiate pro‐oxidant activity that could reduce the overall reduction in antioxidant activity. One reaction that can occur during heating is the non‐enzymatic Millard reaction which causes both positive and negative effects on the overall bioactivity and antioxidant activity (Tomas et al. 2017). The effect is mostly dependent on food type, the nature of the bioactive compound, method, and the intensity of the thermal process which could increase or decrease or even create a newly identified phenolic compound (Peanparkdee et al. 2020; Rodríguez‐Roque et al. 2015; Rohadi et al. 2021).

As glycation induced the formation of AGE and amyloid cross β aggregation was accelerated by AGE‐mediated crosslinking. The aggregation of amyloid cross β could create fibrillar structures (protein aggregation) which is one of the factors contributing to the development of Alzheimer's disease. AGE is a product produced by a non‐enzymatic reaction, more specifically the glycation process in the presence of protein and reducing sugar. The glycation process consists of 3 main stages, namely the early, intermediate, and advanced stages. In the early stage, the interaction between the amino group of protein and the carboxy group of reducing sugar occurs and produces a Schiff's base structure, which is unstable and transforms into an Amadori product or fructosamine (early stage) (Miroliaei et al. 2011). The fructosamine is further rearranged, dehydrated, oxidized, and polymerized into AGE (advanced stage). In the presence of AGE, the protein structure could be further modified into a β‐sheet structure that could be further processed into an oligomer and then finally into an amyloid cross β structure. This unusual protein aggregation could cause further damage or induce synapse loss between neuron cells, which leads to the risk of Alzheimer's disease (Stefanello et al. 2019).

Numerous studies have reported many bioactive compounds in roasted coffee such as phenolic compounds, caffeine, and melanoidins (Monente et al. 2015). The bioactive compounds from roasted coffee beans or coffee, especially chlorogenic acid (CGA) and derivatives, have been reported to reduce advanced glycation end‐product (AGE) accumulation, which could be referred to as anti‐glycation properties (Herawati et al. 2019). CGA and derivatives could still be present in HSCG and CSCG and thus provide anti‐glycation properties to this by‐product. However, the comparison between the impact of thermal treatment and in vitro digestion on antioxidant activity and anti‐glycation properties of antioxidant crude extract from HSCG and CSCG has not yet been revealed. Thus, in this study, the formation of fructosamine, AGEs, and amyloid cross β structure was determined to evaluate the in vitro anti‐glycation properties of ethanoic crude extract from both HSCG and CSCG after thermal treatment and in vitro digestion.

## 2 Materials and Methods

The CSCG and HSCG were obtained from a private company in Chiang Mai Province, Thailand which was organic shade grow coffee bean ( *Coffea arabica* L.) which harvested between September and March of each year and then incubated at room temperature before light roasting. The beans were roasted and brewed within 1 week prior to being sent to the lab for extraction. The CSCG was obtained from the cold brew technique (4°C, 24 h) while HSCG was obtained from hot brew espresso technique using an espresso machine (Sage Espresso Machine, model: BES875UK, Italia). The CSCG and HSCG were freeze‐dried to prevent microbial growth and kept at −18°C before extraction. The antioxidant crude extract from HSCG and CSCG was prepared according to previous research (Chongsrimsirisakhol and Pirak 2022). Briefly, both SCGs were extracted by the ultrasound‐assisted extraction (UAE) process using a thermosonicator bath with 40 kHz (D6 series, GT SONIC, China) according to Onamon and Pirak, (Chongsrimsirisakhol and Pirak 2022) at 50°C, ethanol 95% ratio 1:20 g/mL for 40 min to obtain an antioxidant crude extract from HSCG and CSCG.

The sample for further analysis was prepared by dissolving 250 μg/mL of antioxidant crude extract from HSCG and CSCG in distilled water and then mixed by a magnetic stirrer for 3 h before collecting the supernatant part after centrifuging at 5000 rpm or 1677 g (Eppendorf centrifuge, Thermo Scientific mySPIN 12, USA.) for 5 min (non‐thermal process/non‐in vitro digestion). These samples were subjected to analyze the impact of thermal treatment and in vitro digestion on antioxidant activity and anti‐glycation properties of antioxidant crude extract.

### 2.1 Determination of Phenolic Profile of Antioxidant Crude Extract From HSCG and CSCG

HPLC‐MS was conducted on a Thermo Scientific Dionex UltiMateTM 3000 RSLC Systems (Thermo Fisher Scientific, Germany) equipped with The SCIEX QTRAP 6500+ High‐Throughput Mass Spectrometer with IonDrive High Energy Detector (AB Sciex LLC, USA). Chromatographic separation was done on an Acclaim RSLC 120 C18 column (120 Å, 2.1 × 100 mm, 2.2 μm) (Thermo Fisher Scientific, Germany). The results were acquired by the SCIEX OS (1.2.6) software (AB Sciex LLC, USA). LC conditions were performed at 30°C, with a flow rate of 0.4 mL/min and an ejection volume of 20 μL. Formic acid in water at 0.1% v/v (solvent A) and formic acid in acetonitrile at 0.1%v/v (solvent B) were used as solvent mobile phase composition. The gradient elution profile of solvent B was as follows: 0.00–3.00 min, 0.0%; 3.00–8.00 min, 0.0%–18.0%; 8.00–15.00 min, 18.0%–35%; 15.00–17.00 min, 35%–90%; 17.00–20.00 min, 90%–0.0%. MS analysis was carried out in negative ionization mode. Tuning of LC/MS was performed prior to the analysis using the serial dilution of target compounds.

### 2.2 Determination of TPC, Caffeine, and Antioxidant Activities

The TPC was determined by the Folin‐Ciocalteau assay as described by previous research (Chongsrimsirisakhol and Pirak 2022). The result was compared with the standard curve of Gallic acid and expressed as mg Gallic acid equivalent (GAE)/ml. The standard solution of caffeine was prepared by dissolving caffeine in 1‐propanol (20 ppm). The λ max was determined by scanning the standard solution between 250 and 300 nm (Thermo Scientific GENESYSTEM 10S UV–Vis spectrophotometer, Hexion, Japan). At this λ max, the standard curve of caffeine (5–50 ppm) was used to determine the caffeine content in the determined sample. Before the determination, the caffeine was extracted from the determined sample by adding 2 mL of saturated sodium chloride (26%) and 5 mL of 1‐propanol to 1 g of a determined sample before leaving the solution to precipitate and filter by filter paper (Whatman No. 1). This caffeine extracted was diluted by 1‐propanol (1:10 w/v) before measuring the absorbance at λ max, and the result was calculated compared to the caffeine standard curve (modified from Murray and Hansen (1995)).

The antioxidant activity of the samples was measured through the DPPH assay. The analysis was performed according to previous research (Chongsrimsirisakhol and Pirak 2022). The results were compared with the Trolox standard curve for DPPH and then expressed as μM Trolox/mL.

### 2.3 Thermal Treatment and In Vitro Digestion Process

The antioxidant crude extract (250 μg/mL) from HSCG and CSCG was prepared in distilled water under a magnetic stirrer for 3 h and then centrifuged at 5000 rpm or 1677 g (Eppendorf centrifuge, Thermo Scientific mySPIN 12, USA.) for 5 min. The supernatant part was collected and then heated in a water bath (Memmert model WB14, Germany) until it reached 72°C for 20 s (pasteurization, Pas) and in an autoclave at 121°C for 15 min using (All American 25 Quart Pressure Canner model 925, USA.) (sterilization: Ster) and then submitted to in vitro gastrointestinal digestion. The digestion was processed according to the INFOGEST standardized method (Brodkorb et al. 2019). First, the oral phase proceeded with a standardized 1:1 (w/w) ratio of sample to simulated oral fluid with salivary amylase. The mixed sample underwent this phase for 2 min at 37°C in a shaking water bath. After 2 min, the sample was transferred to the gastric phase. During the gastric phase, the simulated gastric fluid was preheated at 37°C before being added to the oral bolus in the ratio of 1:1 (v/v) with pepsin solution. The pH was adjusted using a pH meter (Docu‐pH, Satorius, Germany) to pH 3.0 by adding HCL (1.5 M) then further incubated at 37°C for 2 h in the same shaking water bath (Memmert model WB14, Germany). Each sample taken was immediately adjusted to pH 7.0 by NaOH (1 M) to minimize enzyme activity, then centrifuged at 5000 rpm or 1677*g* (Eppendorf centrifuge, Thermo Scientific mySPIN 12, USA) for 10 min. The supernatant was collected and stored at −18°C for further analysis. In the intestinal phaฺse, the pre‐warmed (37°C) simulated intestinal fluid was added to the gastric chyme to the final ratio of 1:1 (v/v) with pancreatic and bile salt solution (10 mM). The pH was adjusted to 7.0 before final incubation for 2 h (37°C) in the same shaking water bath. Each sample taken was immediately adjusted to pH 2.0 using HCl (1.5 M) to minimize enzyme activity, then centrifuged at 5000 rpm or 1677*g* (Eppendorf centrifuge, Thermo Scientific mySPIN 12, USA.) for 10 min; then the supernatant was collected and stored at −18°C for future analysis.

### 2.4 Determination of Anti‐Glycation Properties Including Fructosamine Content, AGE, and the Content of Amyloid Cross β Structure

In this experiment, there were 13 samples subjected to the anti‐glycation analysis, which were 6 samples of CSCG extracts treated with different conditions, 6 samples of HSCG extracts treated with different conditions, and one control sample, which was the treatment without the extract.

The treatments of CSCG extract were (1) CSCG extract without heat treatment (BH), (2) CSCG extract after in vitro gastrointestinal digestion (AD), (3) CSCG extract treated with HTST pasteurization (Pas), (4) CSCG extract treated with sterilization (Ster), (5) CSCG extract that treated with pasteurization process and subjected to in vitro gastrointestinal digestion (Pas_AD) and (6) CSCG extract that treated with sterilization process and subjected to in vitro gastrointestinal digestion (Ster_AD). The HSCG extract were treated similar to CHSCG, and 6 samples were received as (1) HSCG extract without heat treatment (BH), (2) HSCG extract after in vitro gastrointestinal digestion (AD), (3) HSCG extract treated with HTST pasteurization (Pas), (4) HSCG extract treated with sterilization (Ster), 5) HSCG extract that was treated with pasteurization process and subjected to in vitro gastrointestinal digestion (Pas_AD) and (6) HSCG extract that was treated with sterilization process and subjected to in vitro gastrointestinal digestion (Ster_AD). The samples were prepared with the same extract concentration (250 μg/mL) before being submitted to the glycation process. The glycation reaction was prepared in the BSA‐glucose system according to Liu et al. (Liu et al. 2018) with minor modifications. First, the BSA (10 mg/mL) and glucose (0.5 M) were prepared separately in the phosphate buffer saline (PBS) (1 M, pH 7.4). The sodium azide (0.5 mM in 1 M PBS, pH 7.4) was also prepared as an anti‐microbial reagent. The BSA, glucose, PBS, and sodium azide were mixed in the ratio of 1:1:2.5: 0.5 v/v, respectively, which represents a normal glycation process (control). The antioxidant crude extract from HSCG and CSCG were mixed in the glycation system by replacing the PBS to achieve the final concentration of 71.43 μg/mL.

The recovery of supernatant of the in vitro digestion treatment after finishing the intestinal phase was performed, and the supernatant was evaporated before being extracted by 95% ethanol at a 1:10 g/mL ratio under a magnetic stirrer at room temperature for 1 h. The liquid extracts were concentrated to dryness through an evaporation process and stored at −18°C before being submitted to the glycation process (modified from Spínola et al. (2020)).

The blank sample was prepared by diluting the supernatant of the antioxidant crude extract with PBS (1 M, pH 7.4) at a 1: 2.5 v/v ratio. The control blank contained only the BSA solution. All the prepared treatments were incubated at 37°C for 21 days under a shaking water bath (100 rpm) (Memmert model WB14, Germany) before determining fructosamine content, AGE, and the content of amyloid cross β structure.

#### 2.4.1 The Determination of Fructosamine Content

The fructosamine developed during the glycation process was analyzed by Nitro‐blue tetrazolium (NBT) assay (Islam et al. 2014). Briefly, the assay starts by mixing the aliquot after 21 days of incubation in NBT solution at a 1:10 v/v ratio, then incubating at 37°C for 15 min using an incubator (NuAire model NU‐5810E, USA.) and reading the absorbance at 530 nm (Thermo Scientific GENESYSTEM 10S UV–Vis spectrophotometer, Hexion, Japan). The solution without adding NBT was prepared as a background sample. The fructosamine content was analyzed compared to the 1‐deoxy‐1‐morpholino‐fructose (1‐DMF) standard curve. The result was reported as IC50 of %inhibition after subtracting the background absorbance (aliquot after 21 days of incubation without added NBT solution).

#### 2.4.2 The Determination of AGE

The aliquot after 21 days of incubation was collected and then measured the fluorescence intensity (FI) through excitation (350 nm) and emission (450 nm) wavelength (FluoroMax + spectroflurometer, Horiba Scientific, USA) which measured AGE fluorescence compound (pentosidine). FI blank was subtracted from all reaction mixtures, and the fluorescent of AGE formation was evaluated and then calculated as an IC50 of % inhibitor (Liu et al. 2018).

#### 2.4.3 The Determination of Amyloid Cross β Structure Content

Thioflavin T was the marker for the amyloid cross β structure that was used to evaluate the changes in protein structure following the glycation process. The assay was prepared according to Liu et al. (2018) with minor modifications in sample preparation. In brief, the thioflavin T solution (64 μM) was mixed with an aliquot of the sample after 21 days of incubation and then left for a reaction at room temperature for 1 h. The FI was measured after 1 h at 435 nm and 485 nm for excitation and emission, respectively (FluoroMax + spectroflurometer, Horiba Scientific, USA). The IC50 was calculated after subtracting the background FI (aliquot after 21 days of incubation without added thioflavin T) based on % inhibitor.

### 2.5 Statistical Analysis

The statistical significance of the variable was determined at the 5% probability level (*p* ≤ 0.05). Analysis of Variance (ANOVA) is used to test the differences of sample mean values with Duncan's New Multiple Range Test at the 95% confidence level using the SPSS program Version 12 (SPSS Inc., USA.).

## 3 Results and Discussion

### 3.1 The Phenolic Profile of Antioxidant Crude Extract From HSCG and CSCG

**FIGURE 1:**
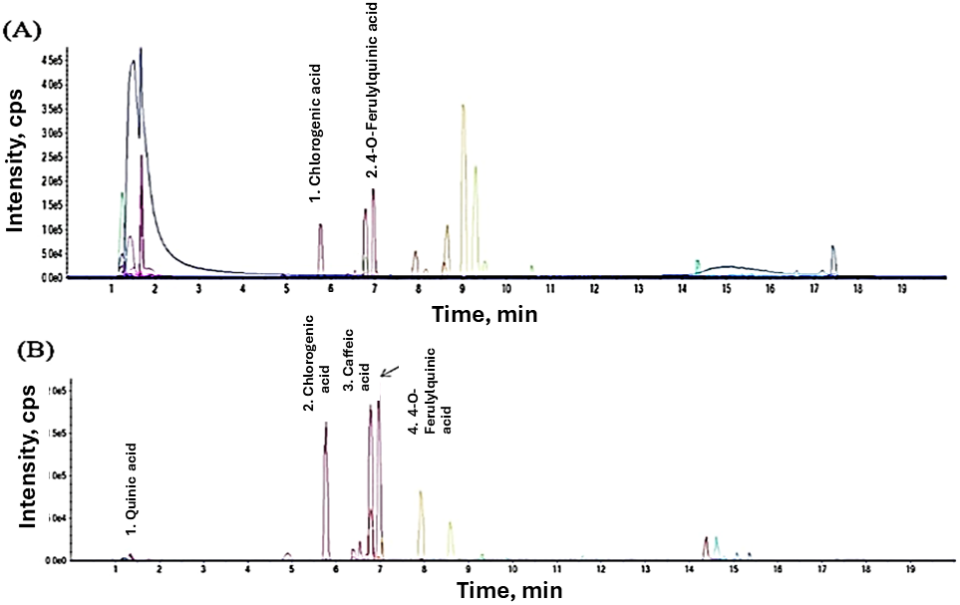
The chromatogram of antioxidant crude extract from HSCG (A) and CSCG (B).

The LC–MS chromatogram of antioxidant crude extract from HSCG and CSCG is shown in Figure 1, and the retention time of each phenolic compound was interpreted as shown in Tables 1 and 2. The antioxidant crude extract from HSCG, 2 bioactive compounds, was found to be neochlorogenic acid (5‐CQA) and 4‐O‐Feruloylquinic acid (4‐FQA). The antioxidant crude extract from CSCG chromatogram showed 4 types of phenolic compounds: quinic acid, 5‐CQA, 4‐FQA, and caffeic acid, which were all derivatives of CGA. Monente et al. (2015) reported a similar type of CQA, and derivatives could be observed in the same type of SCG sample from Arabica or Robusta even though the SCG was obtained from different brewing techniques (filter or capsule). The reduced number of bioactive compounds found in the HSCG extract compared to the CSCG extract could be attributed to the different brewing techniques. The higher temperatures used in hot brewing could extract more bioactive compounds from the coffee, leaving bioactive compounds in the HSCG compared to CSCG.

**TABLE 1:** The phenolic compounds in antioxidant crude extract from HSCG.

Peak number	Retention time	Compounds
1	5.77	5‐CQA
2	7.93	4‐FQA

**TABLE 2:** The phenolic compounds in antioxidant crude extract from CSCG.

Peak number	Retention time	Compounds
1	1.23	Quinic acid
2	5.77	5‐CQA
3	6.95	Caffeic acid
4	7.93	4‐FQA

The CGA and their derivatives were mostly observed as bioactive compounds in the SCG as 3 CQA, 5CQA, 4FQA, 5FQA, caffeic acid, and quinic acid. Boyadzhieva et al. (2018), Burniol‐Figols et al. (2016), Mussatto et al. (2011), Panusa et al. (2013) reported finding CGA and derivatives in the SCG, but at different levels. Most likely due to the difference in SCG's origin, brewing technique, and roasting degree, which influence the presence of CGA in SCGs (Castaldo et al. 2021).

A similar finding was reported by many researchers who observed that the CGA and derivatives in roasted coffee (Liao et al. 2022), silver skin (Nzekoue et al. 2020; Wen et al. 2019; Zhang et al. 2021), and SCGs (Acevedo et al. 2013; Angeloni et al. 2020; Bravo et al. 2012; Głowacka et al. 2019). However, other studies found more bioactive compounds in the SCGs than our findings, which could be attributed to the difference in the roasted degree, origin, and type of coffee, extraction method, extraction solvent, or even the sensitivity of the determination equipment.

### 3.2 The Effect of In Vitro Digestion on Total Phenolic Content and Antioxidant Activity of Antioxidant Crude Extract From HSCG and CSCG

**FIGURE 2:**
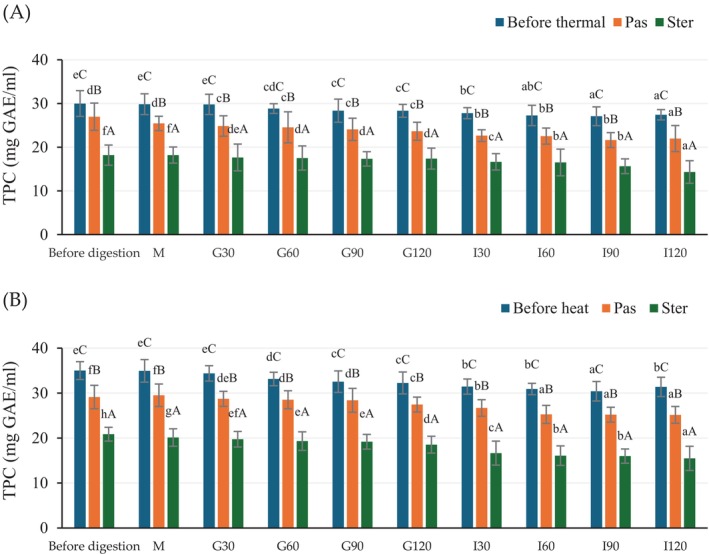
The TPC of antioxidant crude extract from HSCG (A) and CSCG (B) during in vitro gastrointestinal conditions. Results were expressed as mean ± SD from duplicate experiments. The difference in lower case letters (a–f) indicates statistically different (*p* ≤ 0.05) within the same treatment while the difference in upper case letters (A–C) indicates statistically different (*p* ≤ 0.05) between treatment samples (before thermal, Pas, and Ster) at the same in vitro gastrointestinal period, respectively.

The TPC result of before and after in vitro gastrointestinal condition was shown in Figure 2. Before the in vitro digestion process, the highest TPC was found in the non‐thermally treated samples in both HSCG and CSCG extracts. In the oral phase, the TPC of all samples showed no significant difference from the undigested samples. In the gastric phase, the TPC continued to decrease in all samples. The reduction of TPC continued through the intestinal phase. The thermal treatments showed lower TPC during every phase of in vitro gastrointestinal conditions when compared to untreated samples from both antioxidant crude extracts. The lowest TPC was observed in samples after sterilization treatment. At the end of in vitro gastrointestinal conditions, the calculated % TPC bio‐accessibility was 91.4%, 81.5%, and 78.7% in non‐thermal, pasteurization, and sterilization treatments, respectively, for HSCG; with corresponding values of 89.6%, 86.4%, and 74.2% for CSCG, respectively. Thus, the thermal treatments reduced both the TPC content and bio‐accessibility observed in both CSCG and HSCG extracts.

**FIGURE 3:**
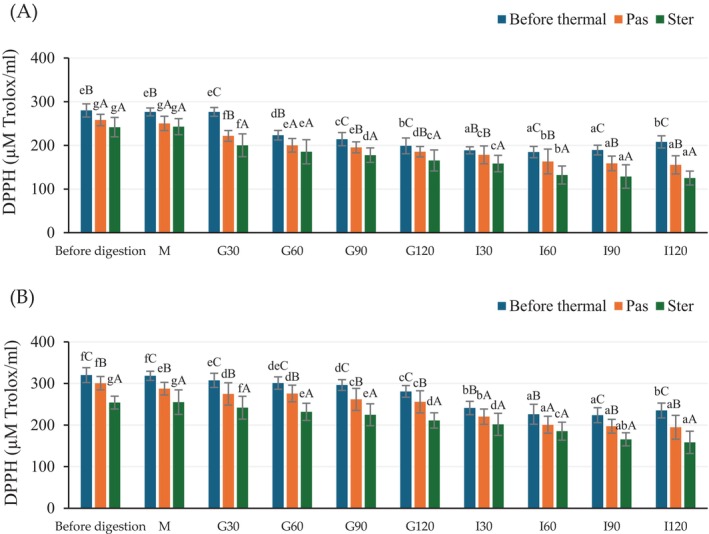
The antioxidant activity (DPPH) of antioxidant crude extract from HSCG (A) and CSCG (B) before and during in vitro gastrointestinal conditions. Results were expressed as mean ± SD from duplicate experiments. The difference in lower case letters (a–f) indicates statistically different (*p* ≤ 0.05) within the same treatment while the difference in upper case letters (A–C) indicates statistically different (*p* ≤ 0.05) between treatment samples (before thermal, Pas, and Ster) at the same in vitro gastrointestinal period, respectively.

The antioxidant activity of the unheated and heated extracts, before and during in vitro gastrointestinal condition, is shown in Figure 3. Similar to TPC, DPPH values of undigested HSCG were lower than the CSCG, and a similar trend resulted. As the oral phase showed no significant effect on the DPPH while during the gastric and intestinal phases, the DPPH value gradually decreased in all treatments. Moreover, thermal treatment lowered the DPPH values when compared to the non‐heat‐treated samples. The DPPH and TPC were dependent and had a high correlation in this study; hence, the analysis of antioxidant activity based on DHHP assay was able to reveal the effect of thermal treatment and in vitro digestion.

**FIGURE 4:**
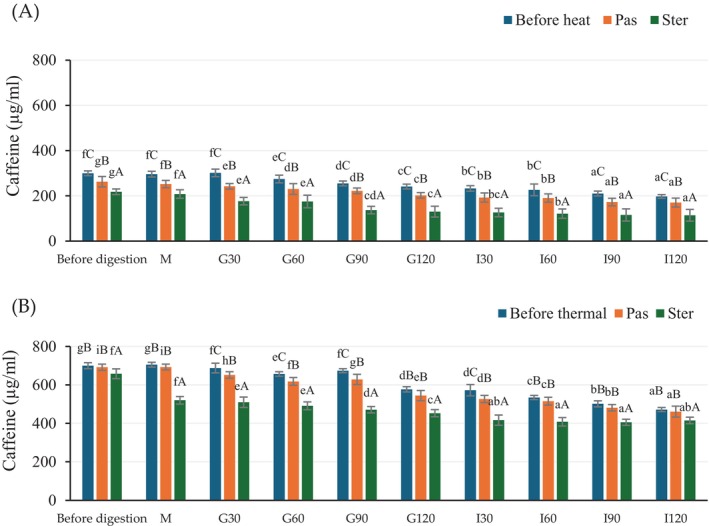
The caffeine content of antioxidant crude extracts from HSCG (A) and CSCG (B) during in vitro gastrointestinal conditions. Results were expressed as mean ± SD from duplicate experiments. The differences in lower case letters (a–f) indicate statistically different (*p* ≤ 0.05) within the same treatment, while the differences in upper case letters (A–C) indicate statistically different (*p* ≤ 0.05) between treatment samples (before thermal, Pas, and Ster) at the same in‐vitro gastrointestinal period, respectively.

The caffeine content of the extracts before and during in vitro gastrointestinal conditions is presented in Figure 4. Before the in vitro digestion process, the highest caffeine content was found in the CSCG extract. Furthermore, heat treatment affected the caffeine content and resulted in a decrease in the content, particularly in the HSCG extract. All samples showed a caffeine content reduction after in vitro gastrointestinal digestion, which was similar to the results of TPC and DPPH (Figures 2 and 3). There was no significant reduction in the oral phase. The caffeine content gradually decreased along with the incubation time for all treatments. The sterilized heat‐treated samples showed a significant reduction of caffeine content which was higher than that of the pasteurized sample. There are conflicting reports on the effect of thermal processing on TPC and antioxidant activity, as treatment could either increase or decrease activity (Guiné and Barroca 2014). According to research from Su et al. (2019) and Rakic et al. (2007) they found an increase in both TPC and antioxidant activity of lychee juice and oak acorn extract after thermal processing. These results could be due to the Maillard reaction promoting the formation of antioxidant compounds such as melanoidin (Choi et al. 2006; Rohadi et al. 2021; Su et al. 2019). In addition, the degradation of auto oxidative enzyme combined with the release of compounds from the softened cell matrix (release of bound phenolics from plant tissue) or the formation of a new phenolic compound through thermally induced reactions (Jeong et al. 2004; Liu et al. 2018). In contrast, other research has reported decreases in both TPC and antioxidant activity after thermal treatment. The reduction was larger with increased temperature and time (Roy et al. 2007). Tomas et al. (2017) and Santhirasegaram et al. (2015) found a reduction in TPC and antioxidant activity of heated tomato (100°C for 60 min) and mango juice (90°C for 60 s) compared to fresh samples. Maghsoudlou et al. (2019) found a remarkable reduction in CGA after thermally treated quince fruit extract.

In this study, the % caffeine bio‐accessibility was calculated, and it was found to be 65.8%, 64.8%, and 52.4% in non‐thermal, pasteurized, and sterilized samples respectively from the HSCG sample; with corresponding values of 67.4%, 66.6%, and 63.1% for the CSCG sample, respectively. The reduction of bio‐accessibility might be due to the degradation of thermo‐labile phenolics and thus a loss in antioxidant activity (Roy et al. 2007; Santhirasegaram et al., 2015). Moreover, high temperature treatment has a negative impact on the TPC and antioxidant activity through the destruction of higher molecular weight polyphenol structure into simple phenolic compounds and induces the degradation of monomeric aglycones to reduce antioxidant activity (Rodríguez‐Roque et al. 2021). Following thermal processing, some bioactive compounds, especially CGA, have been reported to be isomerized or dehydrated and then transformed into other intermediate pro‐oxidant compounds during different stages of the Maillard reaction (Braghini et al. 2019; Kataria et al. 2022; Wu et al. 2022). Moreover, CGA is readily oxidized as the esterified bond of CGA has a high susceptibility to heat and decomposition by the hydrolysis of C‐C and then disposing of the lactone ring from quinic acid which reduces antioxidant activity (Li et al. 2020; Maghsoudlou et al. 2019). The degradation of bioactive compounds was also associated with enzymatic oxidation reactions activated through oxygen or auto‐oxidation that catalyzes the oxidative polymerization of phenolic acid or the basic thermal degradation of naturally occurring antioxidant compounds (Donado‐Pestana et al. 2012; Kim et al. 2021).

In this research, the reduction of TPC, caffeine, and DPPH was observed after HTST pasteurization or sterilization in both HSCG and CSCG extracts. These results indicate that any effect of more bioactive compounds released from the extracted matrix or the formation of a new phenolic compound was less than the heat‐induced degradation or isomerization of phenolic compounds resulting in a reduction of bioactivity. Moreover, the heat processes, either pasteurization or sterilization, significantly affected the stability and bio‐accessibility of the phenolic compounds following digestion. During in vitro digestion, the bioactive compounds go through various structural modifications and degradation, similar to those found for the thermal process (Kumari and Gunathilake 2020). A similar observation was also reported by Sęczyk et al. (2021), Rodríguez‐Roque et al. (2015), Quan et al. (2020), Peanparkdee et al. (2020). After submitting the heat‐treated phenolic extract (CGA) to in vitro digestion, the overall phenolic content decreased compared to the untreated sample (Sęczyk et al. 2021). The vitamin C % bio‐accessibility of heat‐treated fruit juice was reduced compared to their native state (Rodríguez‐Roque et al. 2015). The reduction of TPC and antioxidant activity bio‐accessibility in thermal‐treated fruit juice and Thai rice bran extract has also been reported by Quan et al. (2020) and Peanparkdee et al. (2020), respectively. Coffee leaf extract also had lower caffeine and CGA % bio‐accessibility when transiting from the gastric to intestinal phase during in vitro gastrointestinal (Siddhi 2022).

Thus, the effect of initial extraction conditions, subsequent heat treatment, and in vitro digestion was revealed, and the progressive decreases of TPC, antioxidant activity, and caffeine content resulted from thermal and chemical degradation.

### 3.3 The Anti‐Glycation Properties of Antioxidant Crude Extract From HSCG and CSCG

**FIGURE 5:**
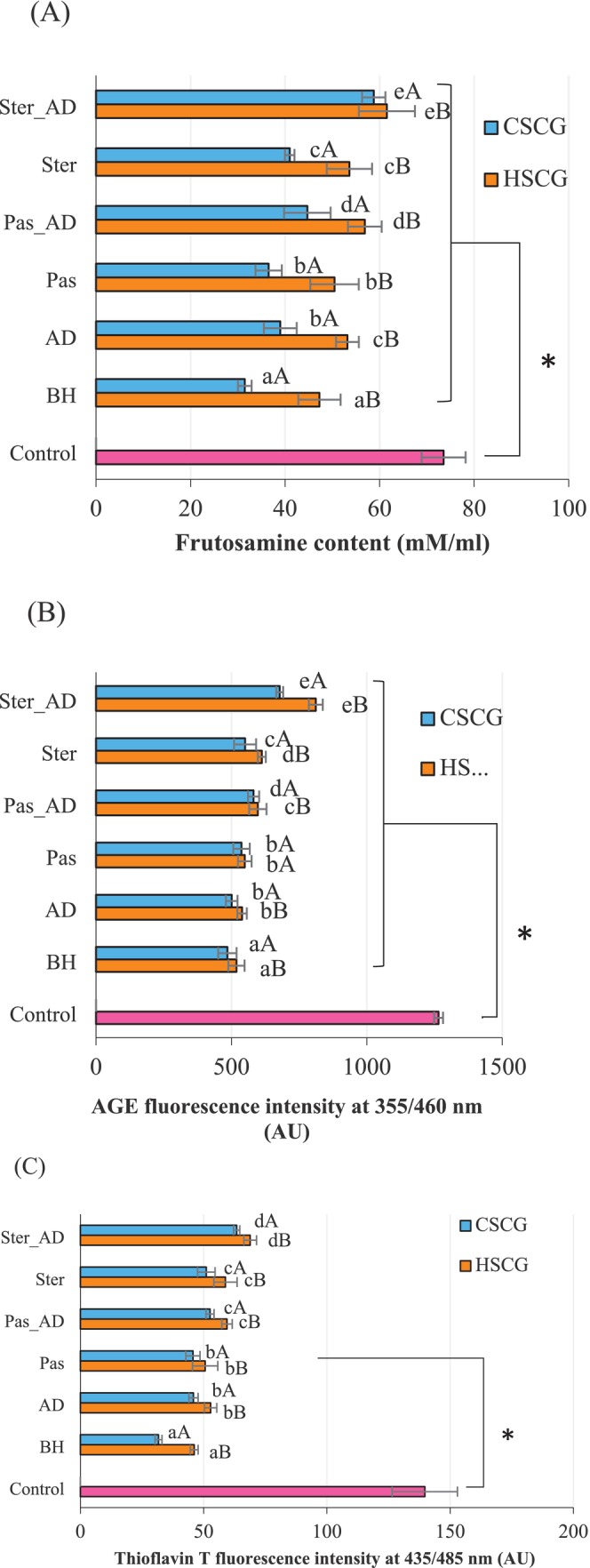
The effect of antioxidant crude extract from HSCG and CSCG on the formation of fructosamine content (A), AGE fluorescence compound (B), and amyloid cross β structure content (C) in the BSA‐glucose system. Results were expressed as mean ± SD from duplicate experiments. The different lowercase letters (a–e) and uppercase letters (A, B) showed a statistical difference at 95% (*p* ≤ 0.05) within the same antioxidant crude extract sample and between the antioxidant crude extract samples, respectively. The * indicates statistically different (*p* ≤ 0.05) between treatment samples (before heat treatment (BH), after in‐vitro gastrointestinal digestion (AD), HTST pasteurization (Pas), and sterilization (ster)), combined with heat treatment and in‐vitro gastrointestinal digestion (Pas_AD and Ster_AD), and control BSA‐glucose system.

In this experiment, the effect of thermal treatment and in vitro digestion on the anti‐glycation properties (fructosamine content, AGE, and amyloid cross β structure content) of 14 samples were revealed Figure 5. The anti‐glycation properties of the HSCG and CSCG extract were evaluated and expressed in terms of fructosamine content, AGE content, and the content of amyloid cross β structure (thioflavin T assay) using the BSA‐glucose system (Wu et al. 2011). It was found that all samples showed significantly lower fructosamine content, AGE content, and amyloid cross β structure content when compared to that of the control (without the extract). This result indicated that the extract either from CSCG or HSCG could inhibit the formation of glycation products. Fructosamine is an early‐stage glycation product while AGE is formed during the advanced‐stage of the glycation reaction. In terms of amyloid cross β structure, this protein structure is modified from α‐helix to beta‐sheet by AGE (Anis and Sreerama 2020; Anwar et al. 2021; Grzegorczyk‐Karolak et al. 2016; Uribarri et al. 2015).

Even though a significant inhibition of glycation products for both HSCG and CSCG extracts was observed, their inhibitory effect was reduced when subjected to thermal treatment and in vitro digestion as presented in Figure 5a–c. The degradation of antioxidant crude extract from CSCG properties in the glycation reaction was mainly attributed to the reduction in TPC and antioxidant activity after the thermal process as shown in the correlation results (Tables 3, 4, 5, 6). The results in Figure 5 showed the effect of heat treatment on the formation of anti‐glycation products after in vitro gastrointestinal digestion. The samples of CSCG and HSCG extracts with and without heat treatment were subjected to the analysis after 3 weeks of incubation (21 days). The fructosamine content of BH and AD samples both from CSCG and HSCG was found to be lower than the heat‐treated samples (Pas, Ster, Pas_AD and Ster_AD) Moreover, the combination of thermal treatment and in vitro digestion (Pas_AD and Ster_AD) resulted in significantly higher (*p* < 0.05) fructosamine content when compared to the samples with thermal process only (Pas and Ster). These results revealed that the heat process could degrade the bioactive compounds and resulted in lowering inhibition effect. Moreover, when subjected to in vitro digestion to mimic the system in the human body, further degradation resulted, and the amount of glycation products was increased (Figure 5a). Similar results were observed in AGE and amyloid cross β structure content. The sample treated with heat and in vitro digestion showed higher (*p* < 0.05) AGE and amyloid cross β structure content compared to the non‐treated samples (BH) (Figure 5b,c). This result might occur because of the bioactive compounds, especially polyphenols, found in CSCG and HSCG extract. These compounds could inhibit or delay the glycation process through many mechanisms, such as the scavenging properties to reduce the generation of the carbonyl group, blocking the reducing sugar carbonyl group, chelation, blocking the Schiff base or Amadori product, and breaking the crosslinking structure AGE from the presence of polyphenol. Moreover, polyphenol could prevent the protein molecule from changing by blocking the protein hydrophobic surface at its substrate side and preventing the structure from refolding into the amyloid fibrils (Miroliaei et al. 2011; Tupe et al. 2016). Hence, the retardation of glycation process was revealed.

**TABLE 3:** The Pearson correlation between TPC, DPPH, fructosamine, AGE, and amyloid cross β structure content of antioxidant crude extract from HSCG after HTST pasteurization.

Parameters	TPC	DPPH	Fructosamine	AGE	Amyloid cross β structure content
TPC	1	—	—	—	—
DPPH	0.641	1	—	—	—
Fructosamine	−0.700*	−0.742*	1	—	—
AGE	−0.456	0.908**	0.955**	1	—
Amyloid cross β structure content	−0.993**	−0.591	0.779*	0.580	1

** *p* ≤ 0.01.

* *p* ≤ 0.05.

The Pearson correlation between TPC, DPPH, fructosamine, AGE, and amyloid cross β structure content of antioxidant crude extract from HSCG after HTST pasteurization was analyzed (Tables 3 and 4). It was found that after pasteurization (Table 3), a significant negative correlation (*p* < 0.05) was found between fructosamine content and TPC and DPPH value. Hence, the increase of TPC or DPPH did not affect the formation of fructosamine. Surprisingly, TPC was found to be significantly negatively correlated (*p* < 0.09) with amyloid cross β structure content at the level of −0.993. Moreover, a significant positive correlation (*p* < 0.01) between AGE and DPPH and AGE and fructosamine was observed at the level of 0.908 and 0.955. These results implied that antioxidant capacity had a significant impact on AGE formation. For the sample treated with sterilization (Table 4), a negative impact was found (*p* < 0.01) between TPC and DPPH with anti‐glycation products. These results indicated that the increase of TPC or DPPH did not alter the changes of anti‐glycation products. The suppression of these compounds might depend on the type of bioactive compounds, not the quantity of the compounds.

**TABLE 4:** The Pearson correlation between TPC, DPPH, fructosamine, AGE, and amyloid cross β structure content of antioxidant crude extract from HSCG after sterilization.

Parameters	TPC	DPPH	Fructosamine	AGE	Amyloid cross β structure content
TPC	1	—	—	—	—
DPPH	0.587	1	—	—	—
Fructosamine	−0.808**	−0.884**	1	—	—
AGE	−0.840**	−0.752**	0.664	1	—
Amyloid cross β structure content	−0.630	−0.666	0.937**	0.883**	1

** *p* ≤ 0.01.

When considering the correlation of CSCG extract after pasteurization (Table 5), the significant positive correlation (*p* < 0.01) between TPC and DPPH resulted with the level of 0.990. However, the TPC was not correlated with fructosamine content (*p* < 0.05) and AGE content (p < 0.01). When considering the correlation of DPPH to anti‐glycation products, it showed similar results to TPC with the significant level of *p* < 0.01. In addition, the TPC of CSCG extract after sterilization was presented in Table 6 indicating the significant positive correlation (*p* < 0.01) of TPC and DPPH, while the negative correlation (*p* < 0.01) was observed between TPC and AGE and amyloid cross β structure content. The negative correlation (*p* < 0.05) between DPPH and fructosamine and AGE content was also revealed. According to the obtained result, it could be assumed that the inhibition of amyloid cross β structure formation was the result of the suppression of both fructosamine and AGE production during the glycation process. As the low fructosamine levels resulted in rection of AGE generation, the subsequent modification of protein structure was then suppressed. Moreover, the high temperature treatment was found to reduce the inhibition of glycation products probably due to the loss or degradation of bioactive compounds, especially for the thermally sensitive compounds by oxidation or non‐enzymatic Maillard reactions (Choi et al. 2006; Kim et al. 2021; Peanparkdee et al. 2020). Furthermore, the non‐enzymatic Maillard reaction could also generate pro‐oxidant compounds in the intermediates of the reaction which further reduces the antioxidant activity following thermal processing. However, this phenomenon depended on the intensity of heat treatment, the nature of the bioactive compound, and the food matrix (Rodríguez‐Roque et al. 2015; Roy et al. 2007; Tomas et al. 2017). Moreover, there was a report describing the loss of CGA and derivatives of quince fruit extract after thermal treatment (Maghsoudlou et al. 2019) as this compound was incompletely degraded by the applied thermal process (Roy et al. 2007). According to the previous discussion, the Maillard reaction could isomerize and dehydrate the CGA and transform the structure into pro‐oxidant compounds (Braghini et al. 2019; Wu et al. 2022). The esterified bond of CGA is very susceptible to oxidation which restructures the lactone ring of quinic acid, reducing antioxidant activity (Li et al. 2020). As CGA and derivatives were the main polyphenol compounds in the antioxidant crude extract from CSCG, then the loss of antioxidant activity and anti‐glycation properties could partly result from the instability or dysfunction of CGA derivatives induced by thermal treatment.

**TABLE 5:** The Pearson correlation between TPC, DPPH, fructosamine, AGE, and amyloid cross β structure content of antioxidant crude extract from CSCG after HTST pasteurization.

Parameters	TPC	DPPH	Fructosamine	AGE	Amyloid cross β structure content
TPC	1	—	—	—	—
DPPH	0.990**	1	—	—	—
Fructosamine	−0.753*	−0.801**	1	—	—
AGE	−0.988**	−0.894**	0.640	1	—
Amyloid cross β structure content	−0.680	−0.568	0.817**	0.756*	1

** *p* ≤ 0.01.

* *p* ≤ 0.05.

**TABLE 6:** The Pearson correlation between TPC, DPPH, fructosamine, AGE, and amyloid cross β structure content of antioxidant crude extract from CSCG after sterilization.

Parameters	TPC	DPPH	Fructosamine	AGE	Amyloid cross β structure content
TPC	1	—	—	—	—
DPPH	0.928**	1	—	—	—
Fructosamine	−0.708*	−0.792*	1	—	—
AGE	−0.831**	−0.746*	0.798*	1	—
Amyloid cross β structure content	−0.905**	−0.682	0.773*	0.795*	1

** *p* ≤ 0.01.

* *p* ≤ 0.05.

## 4 Conclusions

CSCG contained more bioactive compounds (Quinic acid, 5‐CQA, Caffeic acid, and 4‐FQA) when compared to HSCG (5‐CQA and 4‐FQA) as detected by HPLC‐MS. The brewing technique directly affected the CGA structure and content, which might be a limitation for acquiring the waste from the hot brew process, which was the main waste stream from the coffee production company and coffee shops. In this study, CSCG was found to be a suitable raw material for antioxidant extraction. To emphasize the impact of waste utilization, the process for SCGs collection, especially from the cold brew technique (CSCG), is very important since it contains high moisture content and is easy to spoil. The hygienic collection process, along with the suitable pretreatment, is a must to retard microbial growth. The collecting process used in this study was conducted and effectively inhibits microbial growth, prolongs the raw material shelf life, and preserves the bioactive compounds in the SCGs, which will be useful for expanding waste valorization with an environmentally friendly impact in the future.

Moreover, CSCG extracts had higher TPC, antioxidant activity, and caffeine content when compared to the HSCG extract. Thermal treatment of the extracts reduced TPC, antioxidant activity, and caffeine content, and this effect was more significant at high temperatures during the sterilization process. In vitro digestion significantly reduced TPC, antioxidant activity, and caffeine content of the extract. With a combination of thermal treatment and in vitro digestion, the greatest reduction was detected. The CSCG and HSCG extracts showed the inhibitory effect on glycation products and showed a correlation with TPC values. Even though the negative effect of thermal treatments and in vitro digestions on the ability of the extracts to inhibit the formation of glycation products and β amyloid structure was detected, it still exhibited better inhibition than control (without the extract). The caffeine content and specific polyphenols (4‐FQA and 5‐CQA) found in the extracts were in response to this inhibitory effect. These results indicate the potential to use SCGs to create food ingredients or supplements capable of delivering health benefits, particularly the extract from the cold brew process, and suggest the optimal thermal processing temperature below 75°C.

## Author Contributions

**Onamon Chongsrimsirisakhol:** data curation (lead), formal analysis (lead), methodology (equal), validation (equal), visualization (equal), writing – original draft (lead). **Kamolwan Jangchud:** resources (equal), supervision (equal), writing – review and editing (equal). **Peter James Wilde:** supervision (equal), writing – review and editing (equal). **Tantawan Pirak:** conceptualization (equal), funding acquisition (lead), methodology (equal), project administration (lead), resources (equal), supervision (equal), validation (equal), writing – review and editing (equal).

## Conflicts of Interest

The authors declare no conflicts of interest.

## Data Availability

The data that supports the findings of this study are available on request from the corresponding author.
